# Atypical Chromoblastomycosis Presenting as a Large Lesion of the Hand and Forearm Resembling Lupus Vulgaris: A Clinicopathological Case Report

**DOI:** 10.7759/cureus.115302

**Published:** 2026-08-27

**Authors:** Garima Wadhwa, Aneet Mahendra, Sanjeev Gupta, Aditi Dabhra, Nitish Goyal, Charu B Atreja

**Affiliations:** 1 Department of Dermatology, Maharishi Markandeshwar Institute of Medical Sciences and Research, Maharishi Markandeshwar (Deemed to Be University), Ambala, IND; 2 Department of Pathology, Maharishi Markandeshwar Institute of Medical Sciences and Research, Maharishi Markandeshwar (Deemed to Be University), Ambala, IND

**Keywords:** atypical presentation, chromoblastomycosis, deep fungal infection, hand lesion, itraconazole treatment

## Abstract

Chromoblastomycosis is a chronic implantation mycosis caused by melanized fungi, typically affecting rural male workers following traumatic inoculation with soil or vegetative matter. It most commonly involves the lower limbs and is often diagnosed late because of its indolent course and its resemblance to other chronic verrucous dermatoses.

We report the case of a 54-year-old man who presented with a 10-year history of a slowly progressive lesion involving the right hand and distal forearm. The lesion was associated with intermittent pruritus and occasional serous discharge. Clinically, the large plaque over the hand and forearm resembled lupus vulgaris, creating diagnostic difficulty. Histopathology, however, demonstrated characteristic sclerotic bodies, establishing the diagnosis of chromoblastomycosis. The case is notable for the unusual upper-limb involvement, large lesion size, and clinical resemblance to lupus vulgaris. This report highlights the importance of considering chromoblastomycosis in the differential diagnosis of long-standing plaques over exposed upper extremities.

## Introduction

Chromoblastomycosis is a chronic subcutaneous mycosis caused by traumatic implantation of melanized (dematiaceous) fungi, most commonly species of *Fonsecaea*, *Cladophialophora*,* Phialophora*, and *Rhinocladiella*, from soil, wood, or vegetative debris [1,2]. It predominantly affects middle-aged rural men engaged in outdoor or agricultural work and usually presents as a slowly enlarging papule that evolves into nodular, plaque-like, or verrucous lesions over months to years [1,2]. Although the disease may occur on any exposed body part, the lower limbs are affected most commonly, while upper-limb lesions are relatively less frequent and may therefore be overlooked clinically [2,3]. In a global review of 7,740 published cases, lesions were reported on the upper limbs in 1,120 of 5,634 patients with available site data (19.9%), compared with 3,197 of 5,639 patients (56.7%) with lower-limb involvement [4]. Similarly, an Indian review of 169 cases identified upper-limb involvement in 33 of 166 patients with available anatomic data (19.9%), whereas 96 patients (57.8%) had lower-limb disease [2]. Chromoblastomycosis often poses a diagnostic challenge because its clinicomorphologic features can closely simulate other chronic granulomatous dermatoses, such as cutaneous tuberculosis and sporotrichosis, as well as other deep fungal infections, inflammatory dermatoses, and neoplastic lesions, leading to delayed recognition [1,5]. Histopathological identification of muriform or sclerotic bodies remains the defining hallmark of diagnosis [1,5].

We describe an atypical case involving the right hand and distal forearm, notable for its unusual upper-extremity distribution, large lesion size, long-standing localized course, and close clinical resemblance to lupus vulgaris. 

## Case presentation

**Figure 1:**
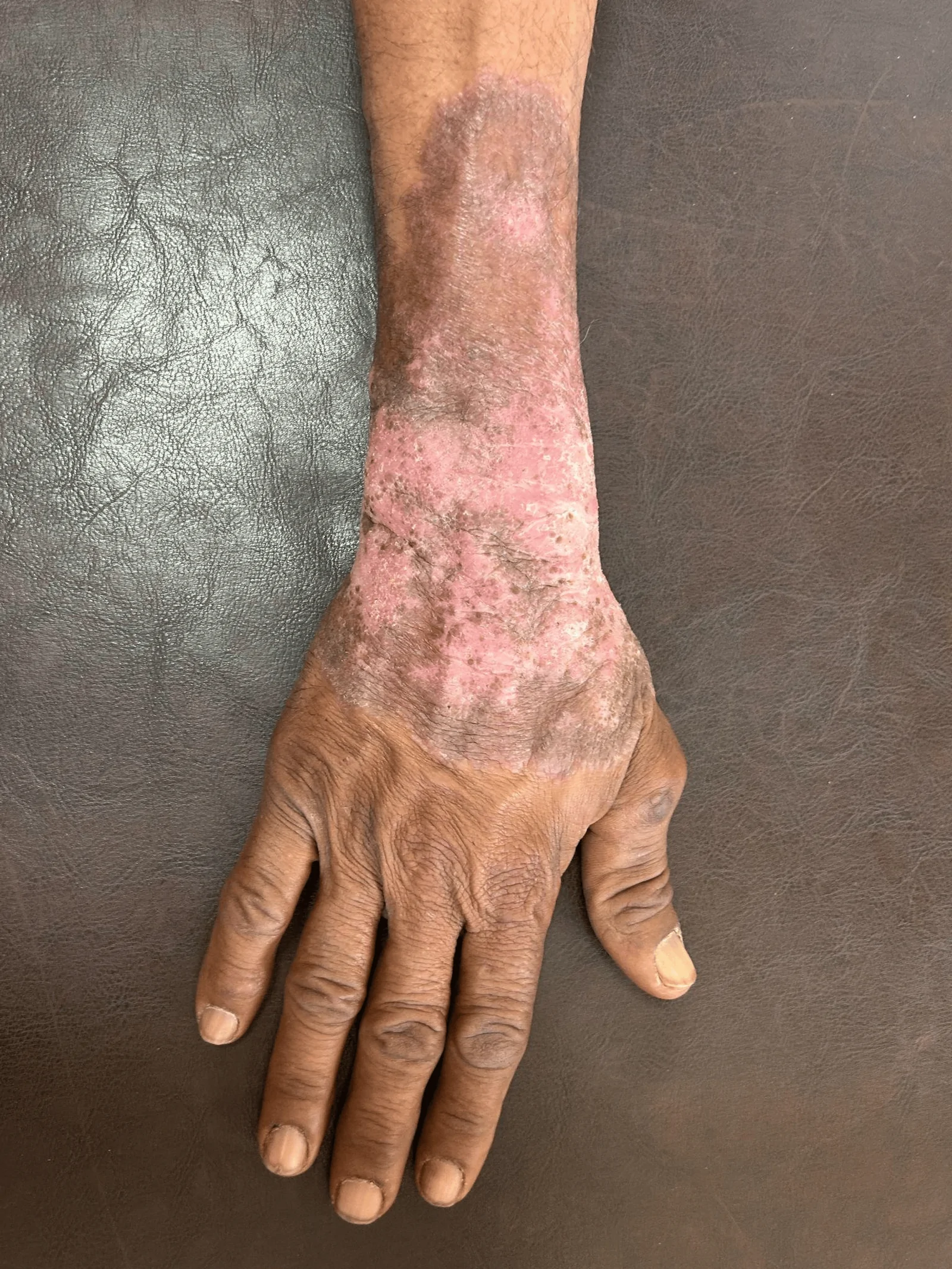
Dorsal right hand and distal forearm showing irregular erythematous to hyperpigmented scaly plaques with mild scaling.

**Figure 2:**
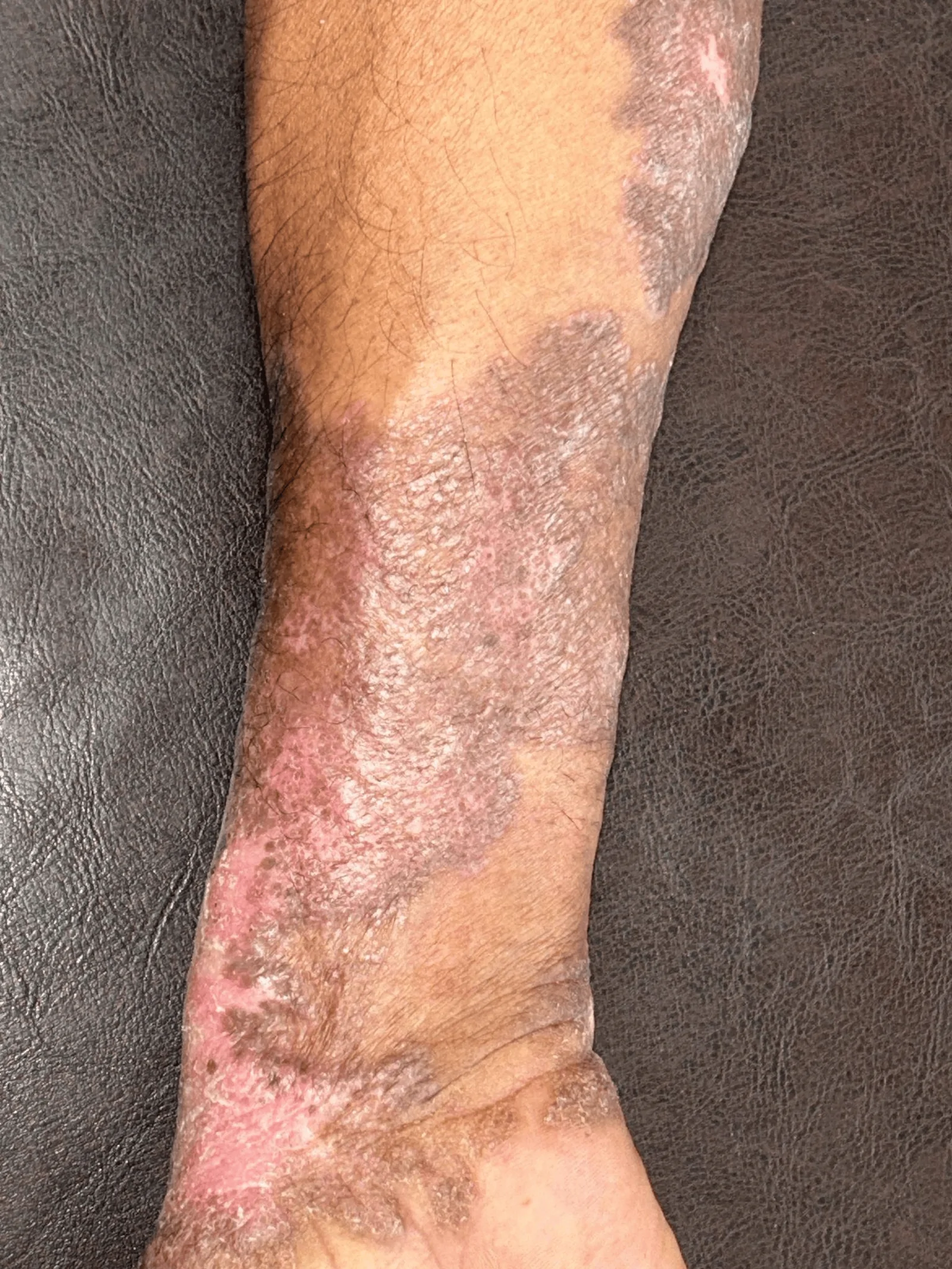
Extension of the lesion to volar aspect of right hand and distal forearm showing irregular erythematous to hyperpigmented scaly plaques with mild scaling.

A 54-year-old man from a rural background presented with a chronic lesion over the right forearm of approximately 10 years’ duration. The lesion began as a small, pea-sized papule following local trauma caused by a wooden splinter during outdoor manual work. Over the next seven to eight years, the lesion gradually enlarged and evolved into irregular, hyperpigmented to erythematous, mildly elevated, scaly plaques over the dorsal and extensor aspects of the right hand and distal forearm, reaching its present size of approximately 14 cm × 5 cm (Figures 1-2). The lesion was associated with intermittent itching of mild-to-moderate intensity and occasional serous discharge. The patient had sought treatment intermittently from a local practitioner, with no significant improvement despite the use of various topical medications.

The lesion showed chronic progressive enlargement with an erythematous to hyperpigmented, scaly morphology, raising a clinical differential diagnosis of lupus vulgaris. There was no history of diabetes mellitus, hypertension, bronchial asthma, or other major systemic illness, and there was no family history of similar lesions. Routine blood investigations were within normal limits. Viral markers were non-reactive. Other baseline laboratory parameters, including lipid profile and thyroid profile, were reportedly normal (Table 1).

**Table 1:** Summary of investigations for the patient. HPF, high-power field; g/dL, grams per deciliter; fL, femtoliter; pg, picogram; U/L, units per liter; mg/dL, milligrams per deciliter; mL, milliliter; mmHg, millimeters of mercury; µL, microliter; ng/dL, nanograms per deciliter; µIU/mL, micro-international units per milliliter

Category	Parameter	Result	Unit	Reference value/range
Vitals	Temperature	37	°C	36.1-37.2
	Pulse rate	88	beats/min	60-100
	Respiratory rate	20	breaths/min	12-20
	Blood pressure	118/72	mmHg	90/60-120/80
Complete blood count	Hemoglobin	14.1	g/dL	13.0-17.0
	Total leukocyte count	8.9	×10³/µL	4.0-10.0
	Neutrophils (polymorphs)	55	%	40-75
	Lymphocytes	33	%	20-45
	Eosinophils	2	%	1-6
	Monocytes	10	%	2-10
	Basophils	0	%	<1
	Red blood cell count	4.83	million/µL	4.50-5.50
	Packed cell volume	43.8	%	36-47
	Mean corpuscular volume	90.7	fL	83-98
	Mean corpuscular hemoglobin	29.2	pg	27.0-32.0
	Mean corpuscular hemoglobin concentration	32.2	g/dL	31.5-34.5
	Platelet count	313	×10³/µL	150-400
Biochemistry	Random blood sugar	79	mg/dL	60-140
	Creatinine	0.70	mg/dL	0.7-1.2
	Aspartate aminotransferase	27	U/L	5-35
	Alanine aminotransferase	31	U/L	5-35
Serology	Viral markers	Non-reactive	-	Non-reactive
Lipid profile	Total cholesterol	178	mg/dL	<200
	Triglycerides	124	mg/dL	<150
	High-density lipoprotein cholesterol	52	mg/dL	≥40 (men); ≥50 (women)
	Low-density lipoprotein cholesterol	98	mg/dL	<100
	Very-low-density lipoprotein cholesterol	25	mg/dL	5-30
	Non-high-density lipoprotein cholesterol	126	mg/dL	<130
Thyroid profile	Free T3	3.2	pg/mL	2.3-4.2
	Free T4	1.3	ng/dL	0.8-1.8
	Thyroid-stimulating hormone	2.45	µIU/mL	0.4-4.0
Urine routine examination	Volume	30	mL	>1.5
	Color	Pale yellow	-	Pale yellow to yellow
	Appearance	Clear	-	Clear
	Urobilinogen	Normal	-	Normal (<0.1 mg/dL)
	Blood	Negative	-	Negative
	Bilirubin	Negative	-	Negative
	Ketone bodies	Negative	-	Negative
	Proteins	Negative	-	Negative
	Glucose	Negative	-	Negative
	pH	5.5	-	5.0-7.0
	Specific gravity	1.025	-	1.001-1.035
	Leukocyte esterase	Nil	-	Nil
	Nitrite	Negative	-	Negative
	Red blood cells	Nil	/HPF	0-2/HPF
	Pus cells	2-3	/HPF	0-5/HPF
	Epithelial cells	Nil	/HPF	0-5/HPF
	Casts	Nil	/HPF	Nil/HPF
	Crystals	Nil	/HPF	Nil/HPF
	Bacteria	Absent	-	Absent
Other investigations	Mantoux test	8	mm	Positive if ≥10 mm

**Figure 3:**
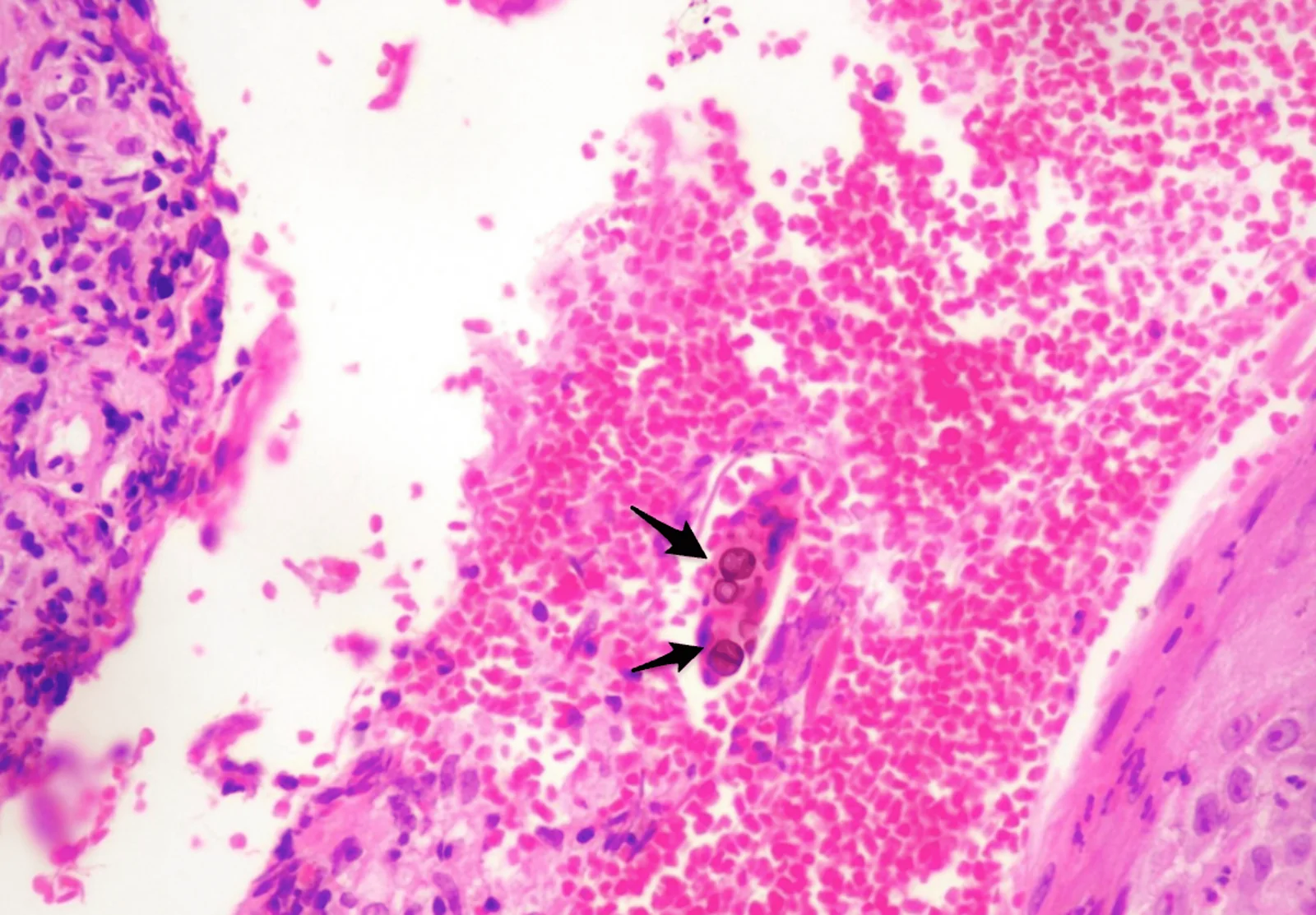
Histopathological examination showing cluster of round to oval, thick-walled, golden-brown sclerotic bodies (muriform cells) depicted by black arrows along with granulomatous inflammatory cells (H&E, ×400).

**Figure 4:**
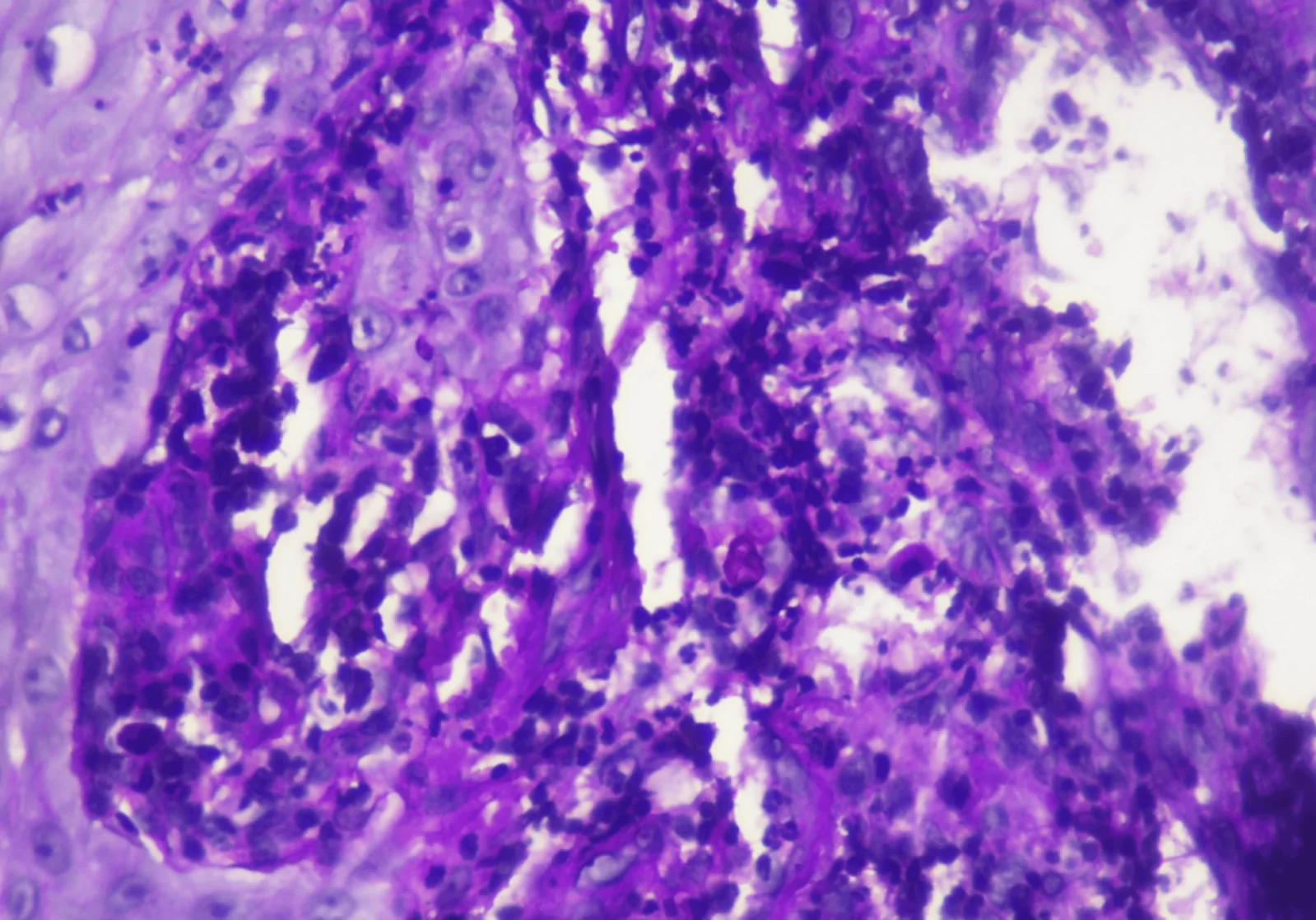
PAS stain showing fungal elements and characteristic brown, thick-walled, muriform (sclerotic or “copper-penny”) bodies. PAS: periodic acid-Schiff

Punch biopsy of the lesional skin from the right forearm was performed, and histopathological examination showed pseudoepitheliomatous hyperplasia of the epidermis with hyperkeratosis. The papillary dermis showed granulomatous inflammation composed of epithelioid histiocytes, multinucleated giant cells, and neutrophilic microabscesses. The surrounding dermis showed a mixed inflammatory cell infiltrate. Few brown, thick-walled spores measuring approximately 4-10 µm, consistent with sclerotic bodies or “copper pennies,” were identified (Figure 3). The histopathological impression was fungal inflammatory pathology with morphological features suggestive of chromoblastomycosis. Periodic acid-Schiff (PAS) staining was performed and highlighted the fungal elements and copper penny bodies, supporting the diagnosis of chromoblastomycosis (Figure 4).

**Figure 5:**
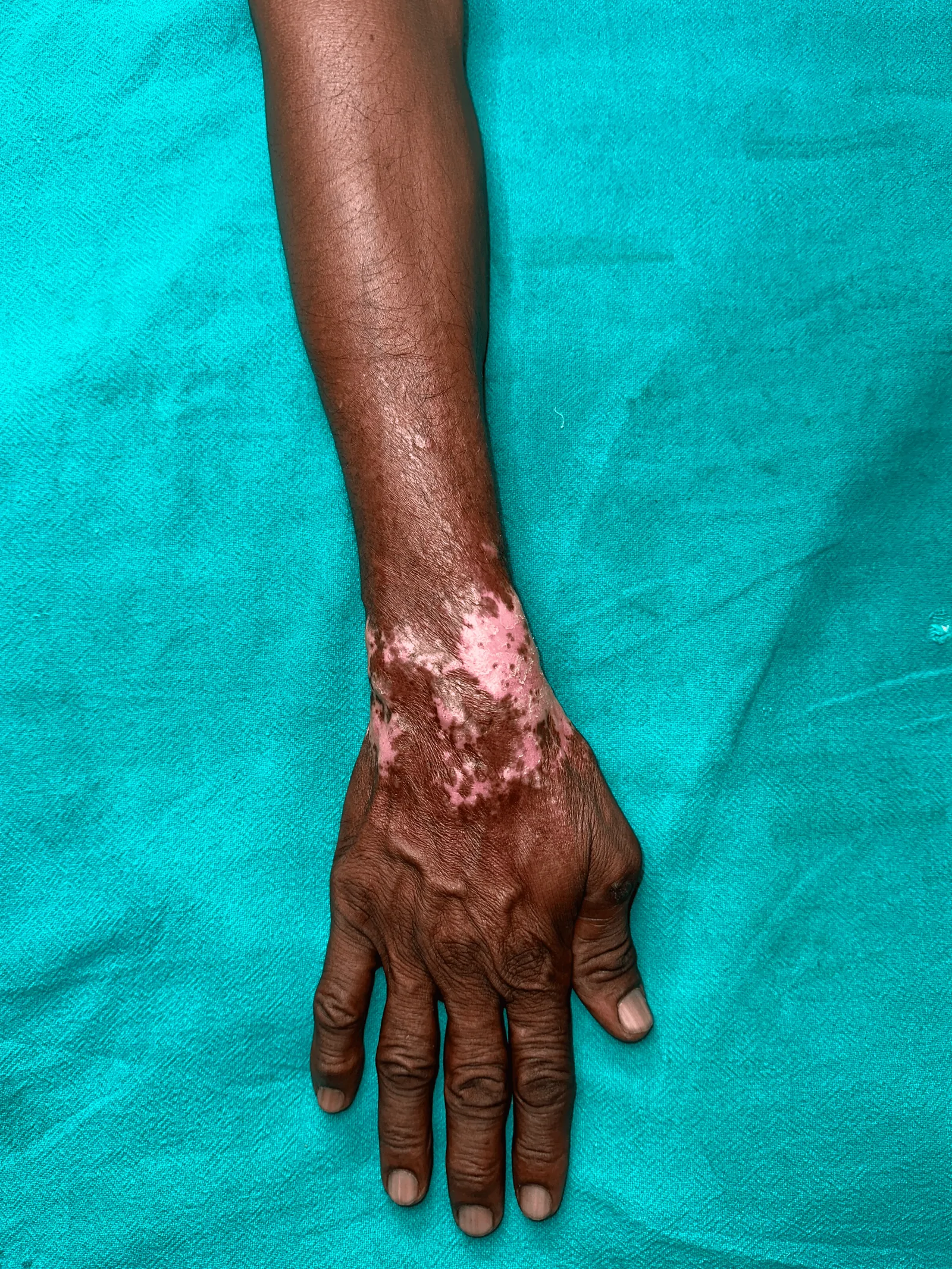
Post-treatment clinical photograph of the dorsal hand and wrist demonstrating marked clinical improvement.

Based on the clinicopathological correlation, a diagnosis of chromoblastomycosis of the right forearm was made. The patient was started on oral itraconazole 200 mg twice daily, with regular monitoring of liver and renal function. Saturated solution of potassium iodide (SSKI) was initiated at eight drops three times daily and gradually increased to 40 drops three times daily. Treatment is ongoing. Marked clinical improvement was observed after three months of therapy, and the patient remains under regular follow-up (Figure 5).

## Discussion

Chromoblastomycosis classically follows traumatic implantation of melanized fungi in rural workers exposed to soil, splinters, thorns, or plant debris [1,2]. In the present case, the history of outdoor manual work, preceding wooden-splinter injury, and indolent progression closely matched the known epidemiologic profile of the disease [1,2].

The atypicality, however, lay in the site, extent, and morphology of the lesion. While lower limbs are affected most often, upper-extremity involvement is distinctly less common [2,3]. Although arm lesions have been described, extensive involvement of the hand and distal forearm remains unusual and may lower initial clinical suspicion, especially when the lesion is localized and slowly progressive [5,6]. In our patient, the lesion remained confined to the right hand and distal forearm for nearly a decade and enlarged to approximately 14 × 5 cm, making it an uncommon and clinically misleading presentation of chromoblastomycosis.

A particularly striking feature was its close clinical resemblance to lupus vulgaris. The large erythematous to hyperpigmented scaly plaque over the exposed hand and distal forearm created a morphologic impression strongly suggestive of lupus vulgaris. This overlap is important, as chronic plaques at exposed sites may easily be attributed to more common granulomatous dermatoses on clinicomorphologic grounds alone. In this case, histopathology proved decisive in establishing the correct diagnosis.

Another notable feature was the prolonged diagnostic delay. Chromoblastomycosis is well recognized for its slow, indolent course and may remain undiagnosed for years because early lesions are nonspecific and later lesions mimic a wide range of inflammatory, infectious, and neoplastic disorders [1,2,6]. In the present case, the lesion had enlarged gradually over many years and had been treated intermittently with topical agents without meaningful improvement, underscoring the importance of early tissue diagnosis in chronic, treatment-resistant erythematous plaques [7].

Histopathological examination revealed pseudoepitheliomatous hyperplasia, granulomatous inflammation with giant cells, neutrophilic microabscesses, and thick-walled brown sclerotic bodies. These findings are characteristic of chromoblastomycosis, and the identification of muriform bodies remains the key diagnostic hallmark of the disease [1,4]. In this patient, clinicopathological correlation was especially crucial because the lesion was morphologically atypical and clinically simulated lupus vulgaris.

The differential diagnosis of a chronic verrucous plaque includes lupus vulgaris, tuberculosis verrucosa cutis, sporotrichosis, blastomycosis, and hypertrophic lichen planus [1,7,8]. Lupus vulgaris and tuberculosis verrucosa cutis may present as chronic plaques on exposed areas and may show epidermal hyperplasia with granulomatous inflammation; however, they require correlation with mycobacterial investigations and do not show muriform bodies [1,7]. Sporotrichosis more typically produces nodules arranged along lymphatic channels, although atypical plaques may occur; yeasts are usually sparse and differ morphologically from the thick-walled muriform bodies of chromoblastomycosis [1,7]. Blastomycosis can also produce pseudoepitheliomatous hyperplasia and suppurative granulomatous inflammation, but broad-based budding yeasts are expected rather than brown sclerotic bodies [1,7]. Hypertrophic lichen planus is a noninfectious mimic and is characterized histologically by a lichenoid/interface inflammatory pattern, hypergranulosis, and keratinocyte injury rather than fungal elements [8]. In the present case, the combination of chronic progression after wooden-splinter trauma and the demonstration of brown, thick-walled muriform bodies favored chromoblastomycosis [1,7].

Upper-extremity chromoblastomycosis is recognized but less frequent than lower-extremity disease. In the Indian review of 169 cases, upper-limb involvement was reported in 33 of 166 patients with available anatomic data (19.9%) [2]. Similarly, a global review identified upper-limb involvement in 1,120 of 5,634 cases with available site data (19.9%) [4]. Pradeepkumar and Joseph reported a nine-year-old boy with a chromoblastomycosis lesion on the left arm that had initially been misdiagnosed as cutaneous tuberculosis; the patient responded well to itraconazole and terbinafine [3]. Pallivalappil and Nair described an atypical forearm presentation in a 48-year-old female rubber tapper, consisting of asymptomatic reddish raised lesions and pustules present for approximately three years; histopathology demonstrated muriform bodies and treatment with itraconazole 200 mg daily was initiated [5]. Falgout and Hilton reported a 1.8-cm papule on the dorsal forearm that had been present for several months; PAS staining supported a deep fungal infection, the lesion was excised, and no recurrence was documented during 18 months of follow-up [6]. A separate report described an 18-year chronic scaly plaque on the auricle of a 65-year-old farmer, emphasizing that unusual anatomical sites can also delay recognition [9]. Compared with these reports, the present case is notable for the 14 × 5 cm lesion. 

Black dots on the surface of a chronic verrucous or hyperkeratotic plaque may represent transepidermal elimination of pigmented fungal elements and can provide an important clinical clue to chromoblastomycosis [7]. When observed, this finding should prompt direct mycological examination of material obtained from the affected surface and histopathological examination; fungal culture may be added when feasible.

According to the clinical severity criteria proposed by Queiroz-Telles et al. [1], mild chromoblastomycosis consists of a solitary plaque or nodule measuring less than 5 cm. Moderate disease consists of solitary or multiple nodular, verrucous, or plaque lesions involving one or two adjacent cutaneous regions and measuring less than 15 cm, whereas severe disease involves extensive cutaneous regions beyond these criteria. The present lesion measured 14 × 5 cm and involved the right hand and distal forearm; therefore, it was classified as moderate chromoblastomycosis

This case carries important teaching value by emphasizing a simple dermatopathologic principle: any chronic, treatment-resistant plaque with verrucous areas over an exposed site in a rural worker warrants early biopsy with careful evaluation for deep fungal infection, particularly when the site and morphology are unusual. Delayed recognition of chromoblastomycosis may lead to fibrosis, secondary infection, lymphedema, and, rarely, malignant transformation [1,9]. The absence of culture and molecular confirmation represents a limitation of this report.

## Conclusions

This case highlights chromoblastomycosis as an important differential diagnosis in chronic plaques over exposed upper extremities. The unusually large lesion involving the right hand and distal forearm, its prolonged localized course, and its close clinical resemblance to lupus vulgaris made the presentation atypical and diagnostically misleading. Early skin biopsy, supplemented by direct mycological examination and fungal culture when feasible, can facilitate timely diagnosis and appropriate management, thereby reducing the risk of chronic complications.
